# 

**Published:** 1889-07

**Authors:** 


### BOOKS AND PAMPHLETS RECEIVED.

"Text Book on Internal Medicine of Domestic Animals Relating to the Diseases of Digestive Organs," by F. Freidberg and E. Ferner. Second edition translated into Russian by J. M. Schulewitch. St. Petersburgh, 1889.

"Thiermedizinische Vortrage. Bd. 1 Heft 7. Halle.

"Studies in the Etiology of the Pneumonia, Complicating Diphtheria in Children," by T. Mitchell Prudden, M.D. and W. P. Northrup, M. D. Reprinted from *Am. Jour. Med. Science.*

"The Operation for Stellate and Bilateral Laceration of the Cervix Uteri," by Hubbard W. Mitchell, M.D. Read before the N. Y. Academy of Medicine, March 24, 1887.

"Fistula in Ano in the Female, operated on by suturing," by Hubbard W. Mitchell, M.D. Read before the N. Y. Academy of Medicine, March 28, 1889.

"Yellow Fever; Absolute Protection Secured by Scientific Quarantine," by Wolfred Nelson, M.D.

"Annual Announcement Cooper Medical College—Session 1889." San Francisco.

"Annual Report of Morse Dispensary for 1888." San Francisco, 1889.

"Annual Report of the Cincinnati Hospital," for the year 1888.

"Annual Report of the Hatch Experiment Station of the Massachusetts Agricultural College,,' January 1889, together with Bulletins Nos. 3–4.

"Bulletin of the Agricultural Experiment Station Cornell University, April 1889."

"Report of the State Board of Health of Illinois." Springfield, 1889.

"Observations upon the Osteology of the North American Anseres," by R. W. Shufeldt, U. S. A. Reprinted from *Pro. U. S. National Museum*, vol. xi.

"Contributions to the Comparative Osteology of Artic and Sub-Artic Water Birds," by R. W. Shufeldt, M.D. Part III. Reprinted from the *Jour. Anat. and Phy.* Vol. xxiii.

"Brief Directions for the Measurement of Small Mammals and for the Preparation of Museum Skins," by C. Hart Merriam, U. S. Dept. Agril. Washington, 1889.

"The Fishery Industries of the United States," by G. Brown Goode. Sec. III–V. Washington, 1887.

"Proceedings United States National Museum, vol. x.

"Fifth Annual Report of the Bureau of Ethnology, 1883–4," by J. W. Powell. Washington, 1887.

AMIENS.—*Bulletin Mensuel Societe Linneenne du Nord de la France.*

AMSTERDAM.—*Bijdragen tot de Dierkunde uitgegeven doo r het K. Zoo. Genootschap.* "Natura Artis Magistra." *Fest-Nummer.* Contains a history of the first fifty years of the society.

AUGSBURG,—*Wochenschrift fur Thierheilkunde und Viehzucht.*

AUGUSTA.—*The Sanitary Inspector.*

BALTIMORE.—*John Hopkins University Circulars.*

BASEL.—*Jahresbericht Zoologischer Garten. 1888.*

BERLIN.—*Archiv fur Wissenschaftliche und Praktische Thierheilkunde.*

BOLOGNA.—*Memorie della R. Accademia delle Scienze dell 'Istituto di Bologna.—Sezione di Medicina e Chirurgia.* Tomo viii. Pt. 3.

BREMEN.—*Abhandlungen Naturwissenschaftlichen Vereine.* Bd. x. Heft 3.

BRISBANE.—*Proceedings of the Royal Society of Queensland.* Vol. 5, Part 1. Contains paper on Filiariae of Birds, by Thomas L. Bancroft.

BRUSSELS.—*Bulletin de l' Academie Royale de Medecine de Belgique.*

BUENOS AIRES.—*Anales de la Sociedad Cientifica Argentina.* Tome xxvi, 4–6, xxvii, 1.

BUFFALO.—*Horse World.*

CALCUTTA.—*Journal of the Asiatic Society of Bengal*, Vol. lvii. No. 4. Contains an article on *Anoplophrya œolosomatis*, a new ciliate infusorian parasitic in the Alimentary canal of *Æolosoma* chlorostictum, by Henry H. Anderson, B.A.

CAP ROUGE.—*Le Naturaliste Canadien.*

CHICAGO.—*The Weekly Drovers' Journal.—National Live Stock Journal.—North American Practitioner.*

CINCINNATI.—*Annual Report of the Zoological Society.*

COPENHAGEN.—*Tidsskrift for Veterinœrer.*

DETROIT.—*The Microscope.—The Pharmaceutical Era.*

DORPAT.—*Sitzungberichte der Naturforscher—Gesellschaft bei der Universitat*, Bd. viii. Heft 3. Contains an article on the formation and growth of the antlers of the European Red Deer, *Cervus elephas*, by Dr. A. v. Mojsisovics.

EDINBURGH.—*Journal of Comparative Pathology and Therapeutics.*

EMDEN.—*72 and 73 Jahresbericht der Naturforschenden Gesellschaft.*

FRANKFORT.—*Der Zoologische Garten.*

GHENT.—*Annales et Bulletin de la Societe de Medecine.*

GRATZ.—*Mittheilungen des Naturwissenschaftlichen Vereines fur Steiermark. Jahr.* 1888.

HARLEM.—*Archives Neerlandaises des Sciences exactes et Naturelles.* Tome xxii, Liv. 2, contains paper on the "Influence of Light on the Purple Bacteria," *Bacterium photometricum* by Th. W. Engelmann.

HAVANA.—*Revista de Ciencias Medicas.*

JENA.—*Jenaische Zeitschrift fur Naturwissenschaft.* Bd. xxiii, Heft 2–3.

KARLSRUHE.—*Thierarztliche Mittheilungen.*

KIEL.—*Schriften des Naturwissenschaftlichen Vereins fur Schleswig-Holstein.* Bd. vii. Heft 2.

KNOXVILLE.—*Agricultural Science.*

LONDON.—*The Veterinary Journal—The Veterinarian.—The Lancet.*

LYON.—*Journal de Medecine Veterinaire et de Zootechnie.*

MADRAS.—*Quarterly Journal of Veterinary Science in India.*

MADRID.—*Gaceta Medico-Veterinaria.*

MILAN.—*La Clinica Veterinaria.*

MONTREAL.—*The Canadian Record of Science.*

NEW YORK.—*Medical Analectic.—New York Medical Journal.—Scientific American.—Forest and Stream.—American Veterinary Review.—Spirit of the Times.—The Sanitarian.—Bnlletin of the American Museum of Natural History*, Vol. ii, No. 2.—*Annual Report of the American Museum*, 1888–9.—*American Naturalist.*

OTTAWA.—*Ottawa Naturalist.*

PALERMO.—*Il Naturalista Siciliano.*

PARIS.—*Le Progress Medical.—Recueil de Medicine Veterinaire.—Bulletin de la Societe Zoologique.* Contains experimental researches on the vermiculose tumors of the Muridæ, by A. Railliet. On the fetus of Needle fish, by Dr. H. E. Sauvage.

PAVIA.—*Bollettino Scientifico.*

PHILADELPHIA.—*Medical and Surgical Reporter—Therapeutic Gazette. Annual Report of the Zoological Society.*

PISA.—*Giornale di Anat. Fisiol. e Patol. degli Animali.*

RED WING.—*Public Health in Minnesota.*

STOCKHOLM.—*Tidskrift for Veterinar-Medicin.*

ST. PETERSBURGH.—*Archives of Veterinary Science.*

STUTTGART.—*Repertorium der Theirheilkunde.*

SYDNEY.—*Proceedings of the Royal Society*, vol. v, part v. Contains a paper on *Aneitea graeffei* and its allies, by C. Hedley.

TORONTO.—*Proceedings of the Canadian Institute.* Vol. vi. Fas. 2. *Annual Report. Session 1887–8.*

TOULOUSE.—*Revue Veterinaire.*

TURIN.—*Bollettino di Musei di zoloogia ed Anatomia comparata. Atti della R. Accademia delle Scienze.* Vol. 23.

UNDINE.—*Giornale di Veterinaira Militare.*

VALENCIA.—*La Cronica Medica.*

VIENNA.—*Œsterreichische Monatschrift fur Theirheilkunde. Annalen des K. K. Naturhistorischen Hofmuseums.* Contains Report on the Ethnology of the South Seas, by Dr. O. Finsch. On Skulls from East Africa, by Dr. A. Weisbach. *Monatschrift des Verines der Thierarzte in Oesterreich—Ornis* v. Heft 1.

WILMINGTON.—*Delaware Farm and Home.*

ZURICH.—*Schweizer Archiv fur Thierheilkunde.*

